# Developing an ensemble machine learning model for early prediction of sepsis-associated acute kidney injury

**DOI:** 10.1016/j.isci.2022.104932

**Published:** 2022-08-12

**Authors:** Luming Zhang, Zichen Wang, Zhenyu Zhou, Shaojin Li, Tao Huang, Haiyan Yin, Jun Lyu

**Affiliations:** 1 Department of Intensive Care Unit, The First Affiliated Hospital of Jinan University, Guangzhou, Guangdong Province 510630, China; 2 Department of Clinical Research, The First Affiliated Hospital of Jinan University, Guangzhou, Guangdong Province 510630, China; 3 Department of Public Health, University of California, Irvine, CA 92697, USA; 4 Warshel Institute for Computational Biology, School of Life and Health Sciences, The Chinese University of Hong Kong (Shenzhen), Shenzhen, Guangdong 518172, China; 5 Department of Orthopaedics, The First Affiliated Hospital of Jinan University, Guangzhou, Guangdong Province 510630, China

**Keywords:** Medicine, Nephrology, Artificial intelligence

## Abstract

Sepsis-associated acute kidney injury (S-AKI) is very common and early prediction is beneficial. This study aiming to develop an accurate ensemble model to predict the risk of S-AKI based on easily available clinical information. Patients with sepsis from the United States (US) database Medical Information Mart for Intensive Care-IV were used as a modeling cohort to predict the occurrence of AKI by combining Support Vector Machine, Random Forest, Neural Network, and Extreme Gradient Boost as four first-level learners via stacking algorithm. The external validation databases were the eICU Collaborative Research Database from US and Critical Care Database comprising infection patients at Zigong Fourth People’s Hospital from China, whose AUROC values for the ensemble model 48–12 h before the onset of AKI were 0.774–0.788 and 0.756–0.813, respectively. In this study, an ensemble model for early prediction of S-AKI onset was developed and it demonstrated good performance in multicenter external datasets.

## Highlights

• We developed an ensemble model to predict sepsis-associated AKI early.
• The model was constructed by stacking algorithm with high discriminative power.
• External tests from US and China showed evidence of generalizability.
• We constructed easy-to-access website calculator for clinicians.

## Graphical abstract

**Figure:**
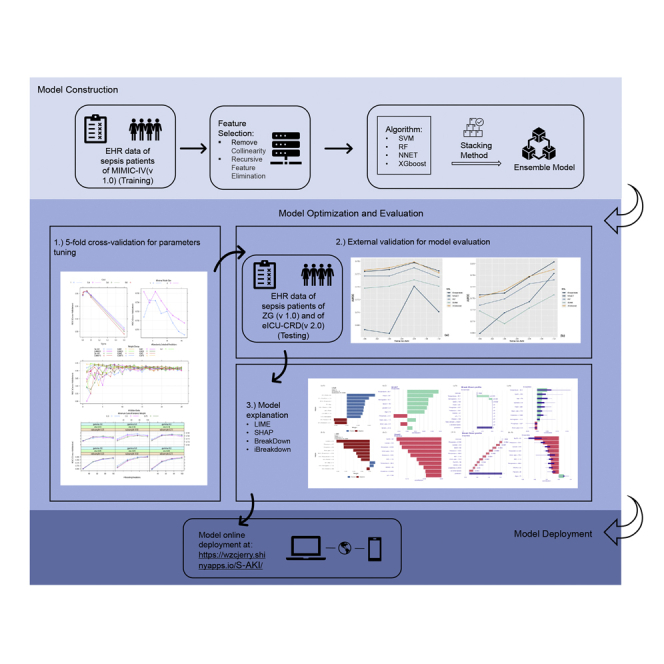

## Introduction

Sepsis is a life-threatening state of organ dysfunction caused by a dysregulated host response to infection (Singer et al., 2016) and represents the leading cause of death for patients in the intensive care unit (ICU) (Hernández et al., 2019). Epidemiologically, sepsis of varying degrees is thought to affect more than 30 million patients worldwide each year, with more than 60% of them dying (Fleischmann et al., 2016). Organ dysfunction, one of the most important features of sepsis, not only increases ICU stay length, hospitalization time, and cost burden for patients but also leads to further deterioration of patient condition and is closely related to poor prognosis (Anderko et al., 2022; Kakihana et al., 2016). A prospective multicenter clinical study from Japan demonstrated that organ dysfunction due to sepsis produces higher mortality and re-hospitalization rates (Fujishima et al., 2014). Acute kidney injury (AKI) is the most common complication in patients with sepsis when organ function is impaired, and the occurrence of more than 50% of AKI cases in ICU units has been reported to be associated with sepsis (Alobaidi et al., 2015). In addition to the adverse effects mentioned above, the appearance of sepsis-associated AKI (SAKI) is also strongly connected with the development of chronic kidney disease (CKD) later in life, as well as an increased risk of long-term mortality (Coca et al., 2009; Kim et al., 2018). A retrospective study including 1,636 patients with sepsis found that approximately 61% of patients developed AKI during hospital admission, and nearly one-fifth of SAKI survivors developed CKD within 1 year of discharge (Arshad et al., 2021). Although scholars have conducted a large number of studies on SAKI so far, effective preventive and therapeutic measures remain lacking. The kidney has a strong reserve function, meaning that by the time creatinine is significantly elevated and urine output is drastically reduced, defined by KDIGO as the diagnostic criteria of AKI (Ostermann et al., 2020), the kidneys have already been damaged to a very serious degree. Therefore, early identification, diagnosis, and intervention of SAKI are of critical importance.

In recent years, machine learning algorithms have become widely used in the medical field. A work by Liu et al. revealed that a machine learning model was better at predicting the risk of surgical site infection in patients after lumbar spine surgery (Liu et al., 2022). Gray et al. have shown that machine learning models outperform logistic regression models in predicting patient prognosis after surgery for colon cancer (Leonard et al., 2022). Researchers have further proposed the concept of ensemble learning (Zhang et al., 2022), which has better performance and generalization ability compared to single machine learning. Zhang developed an ensemble model for predicting agitation in patients with invasive mechanical ventilation under mild sedation. Compared with logistic regression and single machine learning models, ensemble learning models show good performance in independent datasets (Zhang et al., 2021).

The focus of this study is to develop an ensemble model with accurate results, high generalization capability, and sufficient utility to predict the risk of AKI in patients with sepsis based on relatively common and easily available clinical information. To achieve this purpose, this paper integrates models by stacking algorithms in ensemble learning, combining four first-level machine learning algorithms (support vector machine (SVM), random forest (RF), Neural Network (NNET), and Extreme Gradient Boosting (XGboost)) to build an ensemble model that can fully exploit clinical data from patients with sepsis to accurately predict the occurrence of AKI.

## Results

### Baseline characteristics of cohorts

**Figure 1:**
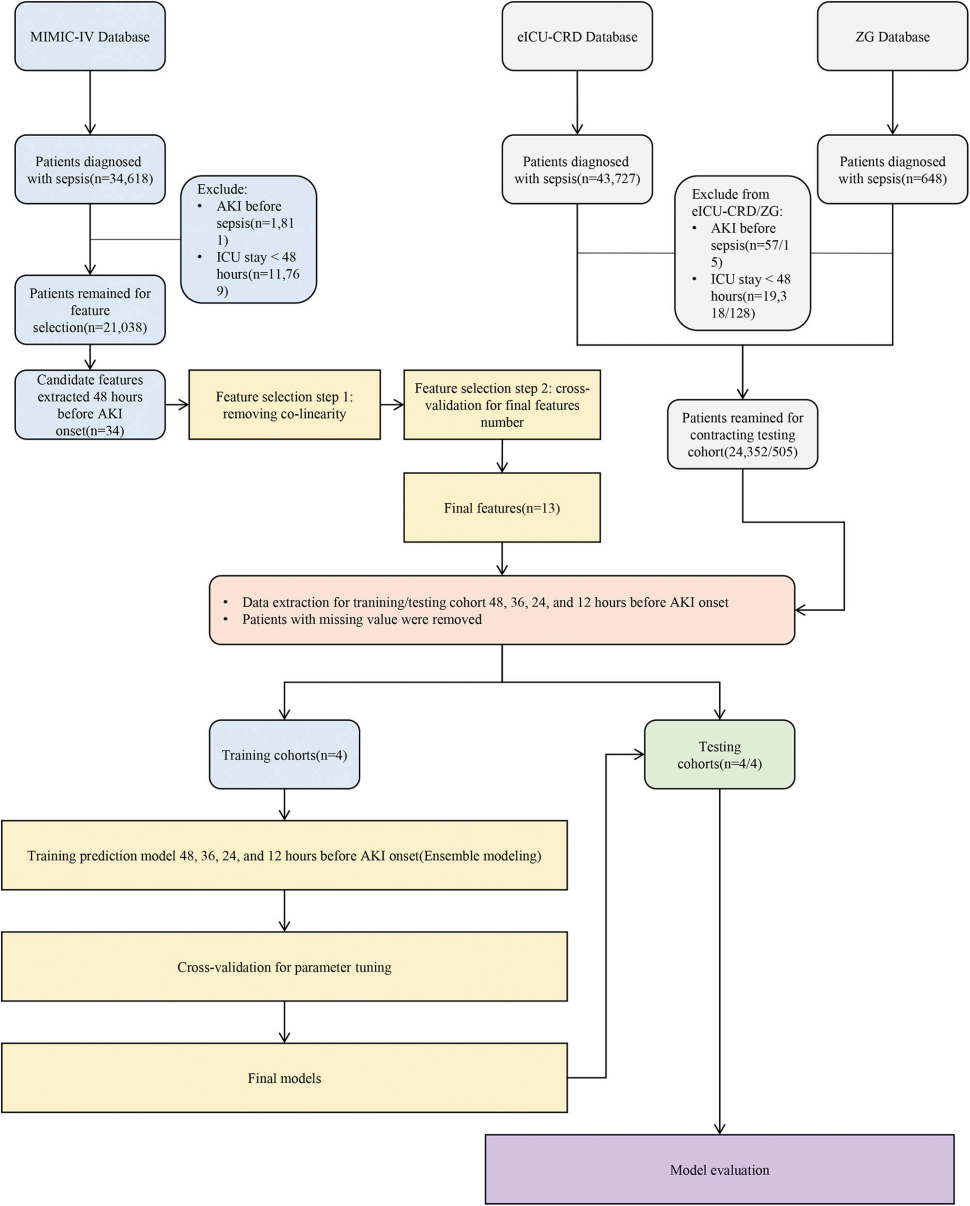
Flow chart for participant inclusion and model processing in the study

**Figure 2:**
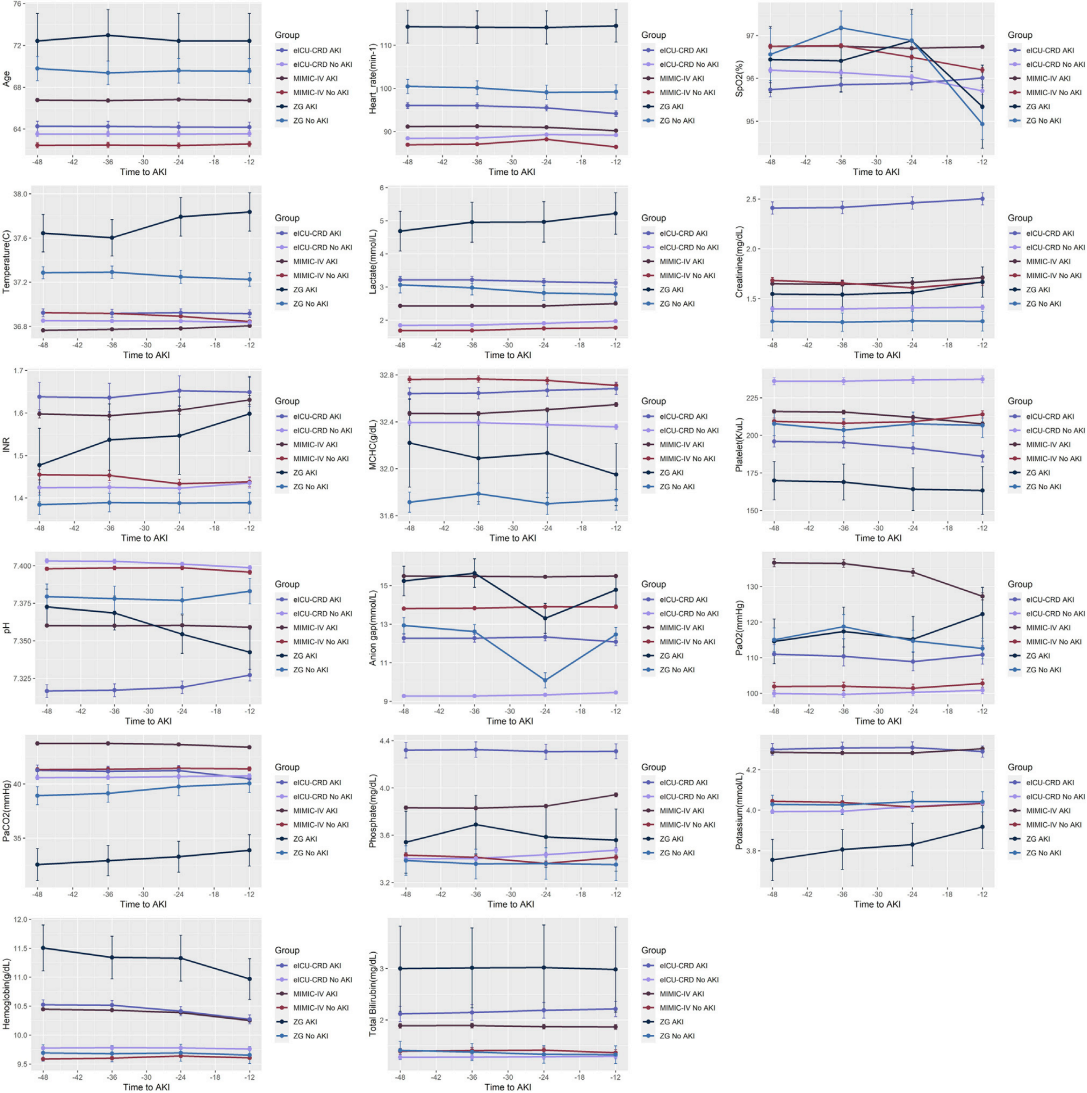
The value of features among datasets 48–12 h before AKI onset For the three databases, the relationship between mean values of features and time before AKI onset was visualized as a line graph; The distance between the error bars and the mean represents SE Although the age of individuals remained constant in the present study, since the composition of the training and testing cohorts are not identical, the mean and SE for each feature’s summary points were different.

Ultimately, 21,038 patients from MIMIC-IV, 24,352 patients from eICU-CRD, and 505 patients from ZG remained for further analysis (Figure 1). Demographic information for MIMIC-IV, eICU-CRD, and ZG patients is shown in (Table 1). The AKI diagnosis rate was 75% in MIMIC-IV patients with sepsis, while the rates for eICU-CRD and ZG were only 25.5% and 20.2%, respectively. Compared to MIMIC-IV and eICU-CRD (12.8% and 10.1%, respectively), the ZG cohort had highest ICU mortality rate (25.0%). Kruskal-Wallis test and Chi-square test demonstrated that the baseline characteristics were significantly different between patients from three databases with high heterogeneity. Longitudinal changes in 17 features over the 48 h before AKI onset are shown in (Figure 2). During this 48-h period, AKI and control groups exhibited good discrimination for most features.

**Table 1:** Baseline characteristics of included sepsis patients from three databases

	MIMIC-IV	eICU-CRD	ZG	p-value
N	21,038	24,352	505	
Age (year)	67 (56, 78)	67(55, 77)	72(62, 81)	<0.001
Gender (%)				<0.001
Male	12,111 (57.6)	12,871 (52.9)	324 (64.2)	
Female	8,927 (42.4)	11,479 (47.1)	181 (35.8)	
Weight (kg)	79.0 (66.4, 95.0)	80.6(65.8, 99.1)	/	/
Height (cm)	170.0 (163.0, 178.0)	168.0 (160.0, 177.8)	/	/
APS	57.0 (42.0, 76.0)	54.0 (40.0, 72.0)	/	/
Unit type (%)				<0.001
MICU/SICU	12,342 (58.7)	19,405 (79.7)	110 (21.8)	
Others	8,696 (41.3)	4,947 (20.3)	395 (78.2)	
Ethnicity (%)				/
White	14,057 (66.8)	18,747 (77.0)	/	
Others	6,981 (33.2)	5,605 (23.0)	/	
Vasopressor (%)				<0.001
No	13,431 (63.8)	18,943 (77.8)	297 (58.8)	
Yes	7,607 (36.2)	5,409 (22.2)	208 (41.2)	
Ventilator (%)				<0.001
No	5,826 (27.7)	7,533 (30.9)	114 (22.6)	
Yes	15,212 (72.3)	16,819 (69.1)	391 (77.4)	
RRT (%)				<0.001
No	20,477 (97.3)	22,896 (94.0)	475 (94.1)	
Yes	561 (2.7)	1456 (6.0)	30 (5.9)	
AKI (%)				<0.001
No	5,253 (25.0)	18,140(74.5)	403(79.8)	
Yes	15,785 (75.0)	6,212(25.5)	102(20.2)	
Length of ICU stay (day)	4.5 (3.0, 8.3)	4.3 (2.9, 7.6)	7.7 (3.9, 16.5)	<0.001
Length of hospital stay (day)	11.0 (7.0, 20.0)	10.5 (6.5, 17.7)	15.8 (6.9, 28.5)	<0.001
ICU mortality (%)				<0.001
No	18,335 (87.2)	21,903 (89.9)	379 (75.0)	
Yes	2,703 (12.8)	2,449 (10.1)	126 (25.0)	

APS: Acute Physiology Score; MICU: Medical Intensive Care Unit; SICU: Surgical Intensive Care Unit; Some of clinical information was not recorded in ZG database therefore replaced by ‘/’.

p-value for continuous variables were calculated by Kruskal-Wallis test and p-value for categorical variables were calculated by Chi-square test.

### Model performance

**Figure 3:**
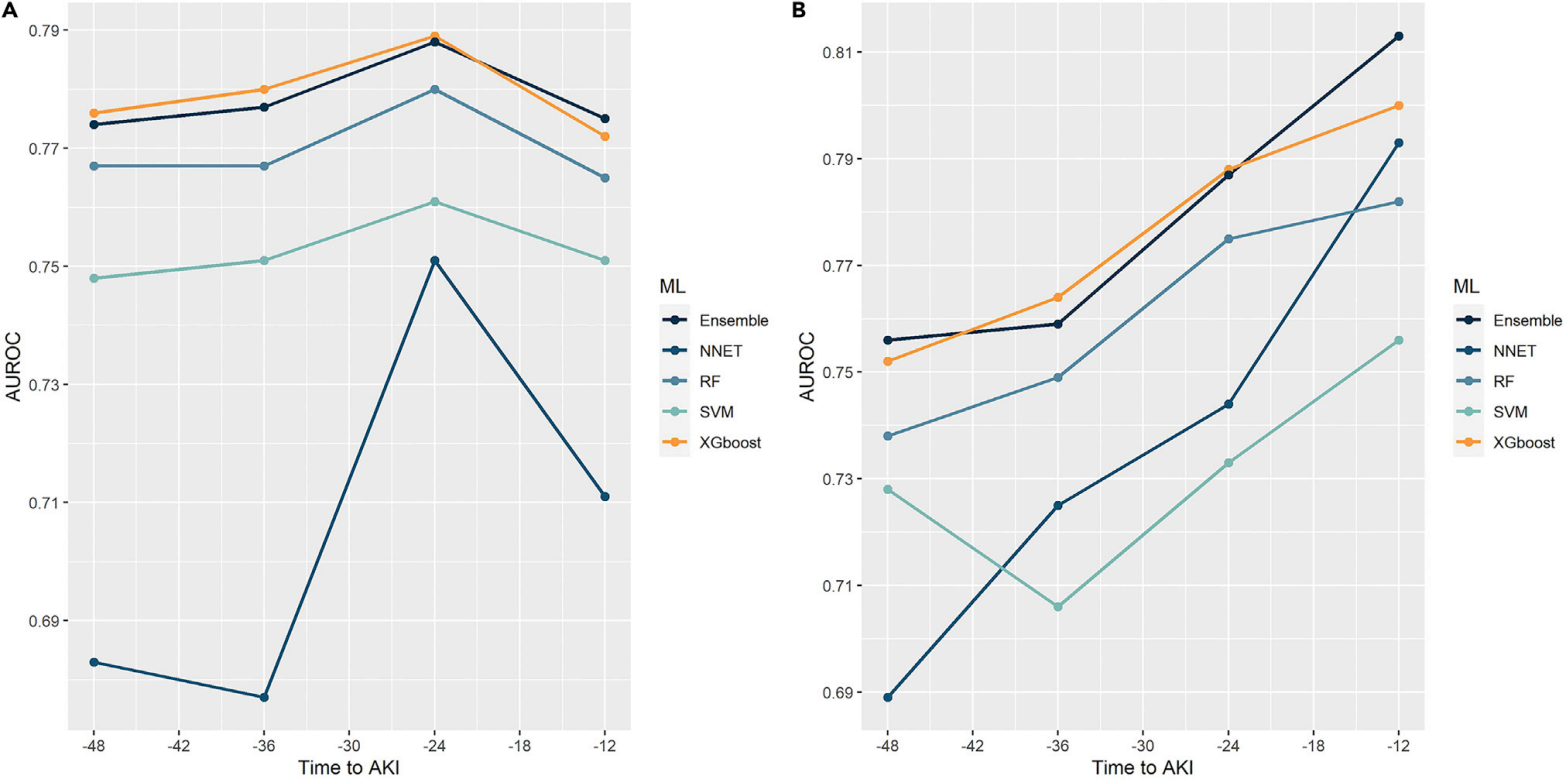
The predictive performance of first-level leaners and the ensemble model AUROC values of four first-level leaners and the ensemble model predicting AKI 12, 24, 36, and 48 h before onset as tested by eICU-CRD datasets (a) and ZG datasets (b).

The performance of models in predicting AKI on test cohorts is shown in (Figure 3). The AUROC values by the ensemble model 48–12 h before the onset of AKI were between 0.774–0.788 and 0.756–0.813 in the eICU-CRD and ZG databases, respectively, indicating good discriminatory capability. The ensemble model’s AUROCs for the first-level learners (SVM, RF, NNET, and XGboost) were in the ranges of 0.683–0.761, 0.765–0.780, 0.677–0.751, and 0.772–0.789, respectively, in the eICU-CRD database and 0.706–0.756, 0.738–0.782, 0.689–0.793, and 0.752–0.800, respectively, in the ZG database. The ensemble model showed the best performance and reached its highest discriminatory capability 12 h before AKI. The performance of ensemble models and related first-level learners in the training cohorts is demonstrated by (Figure S9).

Other performance metrics of the ensemble models for the two testing cohorts are quantified in (Table 2). The sensitivity values of the ensemble model were 0.650–0.724 and 0.685–0.840 for the eICU-CRD and ZG datasets, respectively, indicating that the ensemble model correctly predicted up to 72.4% and 84.0% of AKI cases in testing cohorts. In addition, the balanced accuracy values of the ensemble model were 0.707–0.724 and 0.728–0.778 for the eICU-CRD and ZG datasets, respectively. The ensemble model also reported evaluation metrics for the first-level learners (Tables S4–S7).

**Table 2:** Evaluation metrics of the ensemble model in testing cohorts

Hours to AKI	Sensitivity	Specificity	PPV	NPV	F1	Accuracy	Balanced Accuracy
**eicu-CRD Database**							

48	0.650	0.764	0.412	0.896	0.505	0.741	0.707
36	0.690	0.737	0.400	0.903	0.506	0.727	0.713
24	0.724	0.723	0.400	0.912	0.516	0.724	0.724
12	0.695	0.738	0.404	0.905	0.511	0.729	0.717

**ZG Database**							

48	0.700	0.757	0.398	0.917	0.507	0.746	0.728
36	0.685	0.771	0.411	0.913	0.514	0.754	0.728
24	0.840	0.716	0.408	0.951	0.549	0.740	0.778
12	0.780	0.743	0.415	0.935	0.542	0.750	0.762

PPV: Positive Predictive Values; NPV: Negative Predictive Values; Balanced Accuracy: (Sensitivity + Sensitivity)/2.

### Model explanation

**Figure 4:**
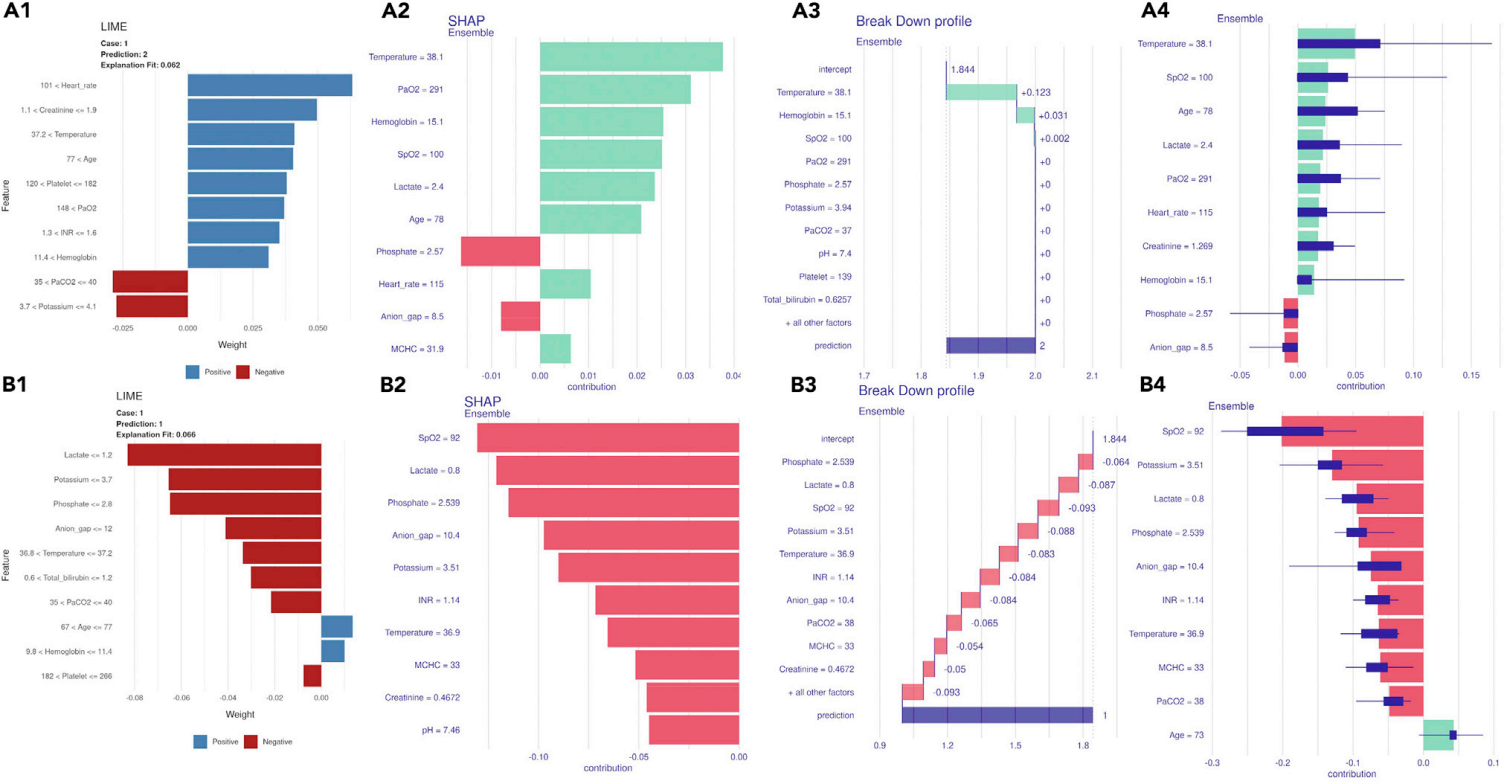
Model explanations for the ensemble model XAI methods for one S-AKI onset and one control patient are exhibited in (a) and (b); (a.1; b.1) represented LIME method; (a.2; b2) represented SHAP method, (a.3; b.3) represented *Break Down* method; (a.4; b.4) represented iBreakDown method. Bar plots to right direction represented positive prediction and bar plots to left direction represented negative prediction. Boxplots for iBreakDown represented the uncertainty of features contributions.

One S-AKI and one control patient from the ZG database were randomly selected as XAI examples. The LIME, SHAP, *Break Down*, and iBreakDown algorithm presented consistent result with slight difference. The LIME method (Figure 4 a.1; b.1) demonstrated that heart rate, creatinine, and temperature made highest contribution for patients with S-AKI while lactate, potassium, and phosphate contributed most for the control patient. The SHAP algorithms result (Figure 4 a.2; b2) showed that for patient with S-AKI, temperature, PaO2, and hemoglobin had most contribution to AKI prediction and for control patient, SpO2, lactate, and phosphate showed most contribution. The *Break Down* results (Figure 4 a.3; b.3) revealed feature contributions to the 12-h ensemble model for one AKI and one control sepsis patient. For the patient with S-AKI, temperature, hemoglobin, and SpO2 were the most important features for positive predicting AKI onset. For the control patient, phosphate, lactate, and SpO2 were the most important features for negative predicting AKI onset. The iBreakDown algorithms resulted (Figure 4 a.4; b.4) suggested that temperature, SpO2, and age contributed most to the probability of positive prediction of S-AKI onset patient and SpO2, potassium, and lactate contributed most to negative prediction of S-AKI onset for the control patient.

### Model deployment

Among the four ensemble models trained on features 48, 36, 24, and 12 h before AKI onset, the XGboost algorithm was the most highly weighted (Figure S8), followed by SVM, RF, and NNET. As mentioned above, to improve running speed and clinical usability, we selected the first-level learner with the most substantial relative influence on the ensemble model, XGboost, and used it to develop an online risk calculator (https://wzcjerry.shinyapps.io/S-AKI/) capable of predicting AKI onset within 12 h for patients with sepsis. In addition, we have attached our ensemble model in the supplementary materials to allow clinicians to download and run the model on their local devices.

## Discussion

In this study, demographic information, laboratory results, and vital signs 48 to 12 h before the onset of SAKI from patients in the MIMIC-IV were collected. To address common problems of medical data, including high dimensionality of features, redundancy of features, and covariance among variables, this study used a two-step feature screening scheme to select feature variables strongly correlated with the occurrence of AKI in patients with sepsis, simplifying model complexity and reducing the negative impact of weakly correlated variables on the prediction model. And four machine learning algorithms subjected to parameter tuning were integrated to maximize efficient disease risk prediction for SAKI. Furthermore, the model was externally validated using EHR data from two other centers in different regions: eICU-CRD and ZG. The results indicated that, in the validated population, the AUROC values predicted by the ensemble model 48–12 h prior to AKI onset were between 0.774–0.788 and 0.756–0.813, respectively, with good discriminatory ability. We also constructed a 12-h online risk calculator based on the XGboost algorithm, which was the most highly weighted first-level learner in the ensemble model, allowing clinicians to calculate the probability of AKI onset within 12 h for patients with sepsis, even without any coding background.

The pathogenesis, clinical manifestations, treatment, and prognosis of AKI are complex and variable. SAKI is further influenced by a variety of factors, including renal blood flow, microcirculation, cortical and medullary perfusion and oxygenation, and renal tubular function (Bellomo et al., 2017). As creatinine and urine volume are not sensitive enough in the early stages of AKI, there have been many published papers exploring how to predict the risk of SAKI more quickly and more accurately. For example, a machine learning model built by Dong et al. can accurately predict the onset of moderate to severe AKI in pediatric wards 48 h before AKI occurrence (Dong et al., 2021). In the last few years, several novel biomarkers have been identified to detect kidney injury and predict the development of AKI. A Chinese emergency cohort study showed that serum cystatin C, kidney injury molecule-1, neutrophil gelatinase-associated lipocalin, klotho, and fibroblast growth factor 23 are valuable in the early prediction of sepsis-related acute kidney injury (Pei et al., 2022).

We conducted four XAI methods for the 12-h ensemble model on two randomly selected patients in an externally validated database, and although there was slight difference in the feature orders, several indicators always ranked high for predicting patients with S-AKI. Temperature, heart rate, hemoglobin, and SpO2 always ranked in the top three contribution in predicting the occurrence of AKI in patients within 12 h, while creatinine, which is associated with AKI diagnostic criteria, interestingly, appeared only once in the LIME algorithm. One potential explanation is that though individuals may appear unregular feature values, the effect on final prediction of the ensemble model is limited since it contained multiple features with complex algorithms. In addition, the XAI result also demonstrated that, for individualized prediction, diagnostic criteria indicator may not always make most important contribution on prediction since in real-world clinics, patients are facing complexing situations which cannot be represented by a single indicator. In patients with sepsis, dysregulation of the infection can lead to an increase in body temperature. Pathogenic microorganisms in the foci of infection, as well as the various toxins released by them, stimulate the release of a large number of inflammatory mediators from monocytes, macrophages, neutrophils, and endothelial cells, which has a direct toxic effect on the renal tubules, leading to renal dysfunction (Schrier and Wang, 2004). While there is evidence to support the idea that early administration of appropriate antibiotic therapy and control of the source of sepsis infection reduces AKI risk, each 1-h delay in antibiotic therapy increases the patient’s risk of AKI by approximately 40% (Bagshaw et al., 2009). Heart rate can be influenced by inflammation, volume status, medications, and interventions, among other factors (Lemm and Buerke, 2022). Hemoglobin and SpO2 can reflect the body’s blood supply and oxygen supply status. Whether this is due to respiratory failure causing hypoxia leading to renal damage or to the hypoperfusion state of the kidney due to insufficient circulating blood volume and the redistribution of renal blood flow, these factors are important in the occurrence and development of SAKI (Ricksten et al., 2021). In non-AKI patient, we can also observe that lactate, which reflects the oxygen metabolism and tissue perfusion status of the body, and SpO2 are always in the forefront as well. Patients with low lactate and well SpO2 are less likely to develop AKI. In the SHAP and Break down results, SpO2 = 100 was a positive predictor of AKI occurrence in patients with AKI, while in non-AKI patients, SpO2 = 92 was a predictor of negative prediction. It should be aware that the XAI result does not mean higher SpO2 is terrible for patients. We can see that the PaO2 of the patient with AKI is 291 mmHg, which is an indication that the patient is being treated with an external supply of oxygen, whether through a nasal cannula or mechanical ventilation. So compared to our randomly selected non-AKI patient, the patient may have a high SpO2, but actually be in a worse respiratory function state. When predicting the probability of AKI in patients with sepsis, the indicators selected by the model need to be analyzed together, rather than focusing on a single one, to arrive at a more accurate probability. As such, in clinical practice, in addition to monitoring creatinine and urine output as noted in diagnostic guidelines, clinicians should also pay comprehensive attention to changes in the above indicators to ensure that appropriate, proactive treatment measures can be initiated, improving the ability to predict and prevent the occurrence of SAKI.

### Limitation of the study

It goes without saying that this paper also has some limitations. Firstly, this paper is a retrospective study, and future prospective experiments are needed to verify the validity of the reported results. Secondly, only four types of machine learning algorithms were used to build the ensemble model, leaving room for further optimization in subsequent research. The principles underlying the stacking algorithm can be analyzed in depth to identify valuable improvements and improve the prediction performance of this model. Finally, the datasets for training and testing were integrated at each timepoint which may lose the richness of the information and may also increase the impact of outliers on prediction to a certain extent therefore further researches based on summarizing data in a period of time or longitudinal modeling are necessary.

### Conclusions

This study developed an ensemble model for predicting the onset of AKI in patients with sepsis. The model exhibited good performance in a multicenter, externally validated dataset.

## Ethics approval and consent to participate

The Medical Information Mart for Intensive Care-IV database was supported by grants from the National Institute of Biomedical Imaging and Bioengineering (NIBIB) of the National Institutes of Health (NIH) under award numbers R01-EB001659 (2003–2013) and R01-EB017205 (2014–2018).

The eICU Collaborative Research Database was released under the Health Insurance Portability and Accountability Act (HIPAA) safe harbor provision. The re-identification risk was certified as meeting safe harbor standards by Privacert (Cambridge, MA) (HIPAA Certification no. 1031219-2).

Critical Care Database comprising infection patients at Zigong Fourth People’s Hospital was conducted in Zigong Fourth People’s Hospital, Sichuan, China from January 2019 to December 2020, and was approved by the ethics committee of Zigong Fourth People’s Hospital (Approval Number: 2020-065).

In these databases, the true identity information about the patient is hidden. Thus, obtaining the patient’s informed consent was not needed. The author completed the relevant course training and obtained the certificate to access these databases.

## Availability of supporting data

All data were downloaded from Physionet (https://physionet.org/) after data permission applications were completed and relevant agreements were signed.

## STAR★Methods

### Key resources table

**Table:** 

REAGENT or RESOURCE	SOURCE	IDENTIFIER
**Deposited Data**		

Medical Information Mart for Intensive Care-IV	Physionet	https://physionet.org/content/mimiciv/1.0/
eICU Collaborative Research Database	Physionet	https://physionet.org/content/eicu-crd/2.0/
Critical Care Database comprising infection patients at Zigong Fourth People’s Hospital	Physionet	https://physionet.org/content/icu-infection-zigong-fourth/1.0/
Structured Query Language	Github	https://github.com/mit-lcp

**Software and algorithms**		

SQL Server (2016)	Microsoft data platform	https://www.microsoft.com/en-us/sql-server/sql-server-downloads
R (v3.6.3)	R CRAN	https://cran.r-project.org/
caret R package (v3.6.3)	R CRAN	https://cran.r-project.org/web/packages/caret/index.html
caretEnsemble R package (v3.6.3) (Ensemble model construction)	R CRAN	https://cran.r-project.org/web/packages/caretEnsemble/index.html
ranger R package (v3.6.3) (Random Forest model construction)	R CRAN	https://cran.r-project.org/web/packages/ranger/index.html
kernlab R package (v3.6.3) (Support Vector Machine construction)	R CRAN	https://cran.r-project.org/web/packages/kernlab/index.html
nnet R package (v3.6.3) (Neural Network construction)	R CRAN	https://cran.r-project.org/web/packages/nnet/index.html
xgboost R package (v3.6.3) (eXtreme Gradient Boosting construction)	R CRAN	https://cran.r-project.org/web/packages/xgboost/index.html
DALEX R package (v3.6.3)	R CRAN	https://cran.r-project.org/web/packages/DALEX/index.html
DALEXtra R package (v3.6.3)	R CRAN	https://cran.r-project.org/web/packages/DALEXtra/index.html
lime R package (v3.6.3)	R CRAN	https://cran.r-project.org/web/packages/lime/index.html
iBreakDown R package (v3.6.3)	R CRAN	https://cran.r-project.org/web/packages/iBreakDown/index.html
dplyr R package (v3.6.3)	R CRAN	https://cran.r-project.org/web/packages/dplyr/index.html
ggplot2 R package (v3.6.3)	R CRAN	https://cran.r-project.org/web/packages/ggplot2/index.html
shiny R package (v3.6.3)	R CRAN	https://cran.r-project.org/web/packages/shiny/index.html

### Resource availability

#### Lead contact

Further information and requests for resources and data should be directed to and will be fulfilled by the lead contact, Jun Lyu (lyujun2020@jnu.edu.cn).

#### Materials availability

This study did not generate new unique reagents.

### Method details

#### Study design and setting

We retrospectively analyzed electronic health records (EHRs) data sourced from the Medical Information Mart for Intensive Care-IV (MIMIC)-IV database (version 1.0): https://doi.org/10.13026/s6n6-xd98 (Johnson et al., 2021), eICU Collaborative Research Database [eICU-CRD (version 2.0)]: https://doi.org/10.13026/C2WM1R (Pollard et al., 2019) and Critical Care Database comprising patients with infection at Zigong Fourth People’s Hospital [ZG (version 1.0)]: https://doi.org/10.13026/gz5h-e561 (Xu et al., 2021).The MIMIC-IV contained over 40,000 ICU admissions from Beth Israel Deaconess Medical Center between 2008 and 2019, and eICU-CRD recorded over 200,000 admissions across 208 United States hospitals between 2014 and 2015(Wu et al., 2021; Yang et al., 2020). The ZG database contained 2,790 infection patients seen between January 2019 and December 2020 at Zigong Fourth People’s Hospital in Zigong, China. EHRs from the three databases were deidentified according to the Health Insurance Portability and Accountability Act (HIPAA) Safe Harbor provision, removing patient name, phone numbers, address, and other potentially identifiable variables from structured data sources. All data were downloaded from Physionet (https://physionet.org/) after data permission applications were completed and relevant agreements were signed.

#### Participants and outcome

All patients (in MIMIC-IV, eICU-CRD and ZG) diagnosed with sepsis according to The Third International Consensus Definitions for Sepsis and Septic Shock (Sepsis-3) were included (Singer et al., 2016). Excluded patients diagnosed with AKI before sepsis onset and patients who stayed in the ICU for less than 48 hours. In this study, EHRs data from MIMIC-IV were selected as the training cohort to construct prediction models which were then externally tested by the eICU-CRD and ZG databases. There was no data overlap between the training and testing cohorts (Figure 1). The outcome of the present study is the onset of AKI (any stage) in sepsis patients according to the definition of the Kidney Disease Improving Global Outcomes (KDIGO)[Increase in serum creatinine by ≥ 0.3 mg/dL (≥26.5 μmol/L) within 48 h; an increase in serum creatinine to ≥1.5 times baseline within the previous 7 days; urine volume ≤0.5 mL/kg/h for 6h](Ostermann et al., 2020). Patients without AKI diagnoses were employed as control groups.

#### Feature selection

We endeavored to build a prediction tool that accurately predicts S-AKI using only data typically generated in the ICU, allowing our model to be more easily implemented in clinics. Therefore, we only included demographic data, laboratory test results and vital signs as potential model features. According to the consensus of three clinical experts, 38 candidate features were extracted from the dataset that 48 hours before AKI onset from MIMIC-IV database of demographic information, laboratory tests, and vital signs, including gender, white blood cells (WBC), red blood cell (RBC), hemoglobin, hematocrit, mean corpuscular hemoglobin (MCH), mean cell hemoglobin concentration (MCHC), mean corpuscular volume (MCV), red cell distribution width (RDW), platelet, anion gap, magnesium, bicarbonate, chloride, sodium, potassium, phosphate, calcium, creatinine, glucose, international normalized ratio (INR), prothrombin time (PT), partial thromboplastin time (PTT), lactate, PaCO2, PaO2, aspartate aminotransferase (AST), total bilirubin, alkaline phosphatase, alanine aminotransferase, pH, albumin, systolic blood pressure (SBP), diastolic blood pressure (DBP), heart rate, respiratory rate, temperature, SpO2 were selected as candidate features. We processed two-step method for feature selection. In the first step, the Pearson correlation coefficients between candidate features were calculated and where correlation >0.7 was considered as collinearity in present study (Figure S2). Based on the suggestion of clinicians, we removed RBC, Hematocrit, MCV, Chloride, PT and AST before step two of feature selection. After removing collinearity, we performed the second step for feature selection by 5-fold cross-validation-based recursive feature elimination (RFE) (Figure S3). According to the RFE result, the model’s accuracy increased from 0.732–0.733 and fell to 0.718 when the number of features (N) was raised to 4. After that, the accuracy of the model gradually increased, reaching its highest point at 0.748 (N = 17). When N exceeded 17, the accuracy of the model fluctuates continuously. Eventually, 17 features [age, anion gap, creatinine, hemoglobin, mean cell hemoglobin concentration (MCHC), phosphate, international normalized ratio (INR), platelet, total bilirubin, potassium, pH, lactate, PaO2, PaCO2, heart rate, temperature and SpO2] were ultimately included.

For AKI onset patients, the event time was AKI diagnosed time and for control group the event time was the ICU discharge time (Cheng et al., 2017). Therefore, Observational windows were spanning 60-12 hours before the event times and features were summarized every 12 hours at the end of each observation windows (Table S1). If there were multiple measurements within 12 hours, the record nearest to the summary time point was selected. Missing values for either training or testing cohorts were removed, leading to non-identical sizes of original dataset, as well as of training and testing data sets for different time points (Table S2).

#### Model construction and evaluation

We constructed an ensemble supervised machine learning model based on the ‘stacking’ method, which refers to fitting multiple machine learning models on the same dataset and using secondary modeling to learn how to best combine their predictions (Shtar et al., 2021). A single sub-model is called a first-level learner, while the combined model is called a second-level learner. In the present study, we combined SVM, RF, NNET and XGboost as first-level learners into our ensemble model. Grid searches were conducted for parameter tunning of all models based on 5-fold cross-validation (Figures S4–S7). Models with highest area under the receiver operating curve (AUROC) in cross-validation were selected as the optimal model and the hyperparameters setting was showed in (Table S3).

Evaluation metrics related to the first- and second-level learners were generated using the external testing cohorts extracted from the eICU-CRD and ZG databases. The optimal threshold of AKI probabilities was used to output a confusion matrix and calculate the AUROC, as well as assess sensitivity, specificity, positive predictive value (PPV), negative predictive value (NPV), F1 score, accuracy and balanced accuracy.

#### Model explanation

For the ensemble model, due to the existence of a “black box,” it is necessary to introduce explanations of the machine learning model (XAI) method. The most common methods in the XAI field that illustrated model behavior on the level of a single prediction are (Local Interpretable Model-Agnostic Explanations) LIME and (SHapley Additive exPlanations) SHAP, and *Break Down* which allow better interpretation of unstructured data but have defects when interpreting tabular data. The idea of the Break Down method is to capture the contribution of a single variable (Y) to the prediction by computing the shift for the expected value of Y while fixing other variable values. If interactions are present, the computed value of the attribution of the Break Down method depends on the order of explanatory covariates that are used in calculations (Staniak and Biecek, 2018). SHAP algorithms based on the idea of averaging variables attribution several numbers of possible orderings, which can be considered as a unification of a collection of different commonly used techniques for model explanations. Compared to SHAP and Break Down, which determine non-zero attributions for all variables, LIME locally approximates a black-box model with simpler sparse explainers, which suits high-dimensional models. The main concept of local explanations, such as SHAP and LIME is showing additive local representations, while complex models are usually non-additive and had inconsistency XAI result (Adak et al., 2022). As a recently developed method, *iBreakDown* algorithm which had similar spirits of SHAP and *Break Down* while not restricted to additive effects, therefore interprets structured data more accurately (Zhang et al., 2022). Furthermore, as a non-additive method, iBreakDown can identify and display feature interactions while showing the uncertainty of the interpretation level. We employed LIME, SHAP *Break Down,* and iBreakDown to explain the ensemble model in present study.

#### Model deployment

We observed that the ensemble model had the highest S-AKI predictive capacity. However, due to its high complexity, the prediction speed of the ensemble model is highly dependent on the hardware used, making it challenging to deploy in a real-world ICU setting. The XGboost algorithm was the most highly weighted first-level learner in the ensemble model and exhibited higher predictive performance than the ensemble model 48-12 hours before AKI onset in the two-testing cohort (Figure S8). Therefore, we built an online risk calculator based on the XGboost algorithm trained on 12 hours of data. Any user can access our online calculator through the website.

### Quantification and statistical analysis

All statistical analyses were performed using R software (The R Project for Statistical Computing), version 3.6.3. Ensemble models were implemented via ‘caret’ and ‘caretEnsemble’ packages. Online risk calculator was developed by ‘Shiny’ package. Descriptive statistics for patients included median (IQR) and counts (percentages) for continuous and categorical variables, respectively. Continuous variables across databases were compared by the Kruskal-Wallis test, and the Chi-square test compared categorical variables. A two-sided p value of <0.05 was considered statistically significant.

## Data Availability

All data supporting the findings of this study can be downloaded from Physionet after completing the data permission application and signing the relevant agreement, detailed in the key resources table. The code to extract data using Structured Query Language can be seen in detail in the official website, detailed in the key resources table. And some examples can be found in the supplementary material (Figure S1). Any additional information required to reanalyze the data reported in this paper is available from the lead contact upon request.

## References

[bib1] Adak A., Pradhan B., Shukla N., Alamri A. (2022). Unboxing deep learning model of food delivery service reviews using explainable artificial intelligence (XAI) technique. Foods.

[bib2] Alobaidi R., Basu R.K., Goldstein S.L., Bagshaw S.M. (2015). Sepsis-associated acute kidney injury. Semin. Nephrol..

[bib3] Anderko R.R., Gómez H., Canna S.W., Shakoory B., Angus D.C., Yealy D.M., Huang D.T., Kellum J.A., Carcillo J.A., ProCESS Investigators (2022). Sepsis with liver dysfunction and coagulopathy predicts an inflammatory pattern of macrophage activation. Intensive Care Med. Exp..

[bib4] Arshad A., Ayaz A., Rehman S., Ukrani R.D., Akbar I., Jamil B. (2021). Progression of acute kidney injury to chronic kidney disease in sepsis survivors: 1-year follow-up study. J. Intensive Care Med..

[bib5] Bagshaw S.M., Lapinsky S., Dial S., Arabi Y., Dodek P., Wood G., Ellis P., Guzman J., Marshall J., Parrillo J.E. (2009). Acute kidney injury in septic shock: clinical outcomes and impact of duration of hypotension prior to initiation of antimicrobial therapy. Intensive Care Med..

[bib6] Bellomo R., Kellum J.A., Ronco C., Wald R., Martensson J., Maiden M., Bagshaw S.M., Glassford N.J., Lankadeva Y., Vaara S.T., Schneider A. (2017). Acute kidney injury in sepsis. Intensive Care Med..

[bib7] Cheng P., Waitman L.R., Hu Y., Liu M. (2017). Predicting inpatient acute kidney injury over different time horizons: how early and accurate?. AMIA Annu. Symp. Proc..

[bib8] Coca S.G., Yusuf B., Shlipak M.G., Garg A.X., Parikh C.R. (2009). Long-term risk of mortality and other adverse outcomes after acute kidney injury: a systematic review and meta-analysis. Am. J. Kidney Dis..

[bib9] Dong J., Feng T., Thapa-Chhetry B., Cho B.G., Shum T., Inwald D.P., Newth C.J.L., Vaidya V.U. (2021). Machine learning model for early prediction of acute kidney injury (AKI) in pediatric critical care. Crit. Care.

[bib10] Fleischmann C., Scherag A., Adhikari N.K.J., Hartog C.S., Tsaganos T., Schlattmann P., Angus D.C., Reinhart K., International Forum of Acute Care Trialists (2016). Assessment of global incidence and mortality of hospital-treated sepsis. Current estimates and limitations. Am. J. Respir. Crit. Care Med..

[bib11] Fujishima S., Gando S., Saitoh D., Mayumi T., Kushimoto S., Shiraishi S.I., Ogura H., Takuma K., Kotani J., Ikeda H. (2014). A multicenter, prospective evaluation of quality of care and mortality in Japan based on the Surviving Sepsis Campaign guidelines. J. Infect. Chemother..

[bib12] Hernández G., Ospina-Tascón G.A., Damiani L.P., Estenssoro E., Dubin A., Hurtado J., Friedman G., Castro R., Alegría L., Teboul J.L. (2019). Effect of a resuscitation strategy targeting peripheral perfusion status vs serum lactate levels on 28-day mortality among patients with septic shock: the ANDROMEDA-SHOCK randomized clinical trial. JAMA.

[bib13] Kakihana Y., Ito T., Nakahara M., Yamaguchi K., Yasuda T. (2016). Sepsis-induced myocardial dysfunction: pathophysiology and management. J. Intensive Care.

[bib14] Johnson A., Bulgarelli L., Pollard T., Horng S., Celi L.A., Mark R. (2021).

[bib15] Kim J.S., Kim Y.J., Ryoo S.M., Sohn C.H., Seo D.W., Ahn S., Lim K.S., Kim W.Y. (2018). One--Year progression and risk factors for the development of chronic kidney disease in septic shock patients with acute kidney injury: a single-centre retrospective cohort study. J. Clin. Med..

[bib16] Lemm H., Buerke M. (2022). Heart rate control in shock. Med. Klin. Intensivmed. Notfmed..

[bib17] Leonard G., South C., Balentine C., Porembka M., Mansour J., Wang S., Yopp A., Polanco P., Zeh H., Augustine M. (2022). Machine learning improves prediction over logistic regression on resected colon cancer patients. J. Surg. Res..

[bib18] Liu W.C., Ying H., Liao W.J., Li M.P., Zhang Y., Luo K., Sun B.L., Liu Z.L., Liu J.M. (2022). Using preoperative and intraoperative factors to predict the risk of surgical site infections after lumbar spinal surgery: a machine learning-based study. World Neurosurg..

[bib19] Ostermann M., Bellomo R., Burdmann E.A., Doi K., Endre Z.H., Goldstein S.L., Kane-Gill S.L., Liu K.D., Prowle J.R., Shaw A.D. (2020). Controversies in acute kidney injury: conclusions from a kidney disease: improving global outcomes (KDIGO) conference. Kidney Int..

[bib20] Pei Y., Zhou G., Wang P., Shi F., Ma X., Zhu J. (2022). Serum cystatin C, kidney injury molecule-1, neutrophil gelatinase-associated lipocalin, klotho and fibroblast growth factor-23 in the early prediction of acute kidney injury associated with sepsis in a Chinese emergency cohort study. Eur. J. Med. Res..

[bib21] Pollard T., Johnson A., Raffa J., Celi L.A., Badawi O., Mark R. (2019).

[bib22] Ricksten S.E., Bragadottir G., Lannemyr L., Redfors B., Skytte J. (2021). Renal hemodynamics, function, and oxygenation in critically ill patients and after major surgery. Kidney.

[bib23] Schrier R.W., Wang W. (2004). Acute renal failure and sepsis. N. Engl. J. Med..

[bib24] Shtar G., Rokach L., Shapira B., Kohn E., Berkovitch M., Berlin M. (2021). Explainable multimodal machine learning model for classifying pregnancy drug safety. Bioinformatics.

[bib25] Singer M., Deutschman C.S., Seymour C.W., Shankar-Hari M., Annane D., Bauer M., Bellomo R., Bernard G.R., Chiche J.D., Coopersmith C.M. (2016). The Third international consensus definitions for sepsis and septic shock (Sepsis-3). JAMA.

[bib26] Staniak, M and Biecek, P, (2018). Explanations of Model Predictions with live and breakDown Packages. R J. 10,2. 10.32614/RJ-2018-072

[bib27] Wu W.T., Li Y.J., Feng A.Z., Li L., Huang T., Xu A.D., Lyu J. (2021). Data mining in clinical big data: the frequently used databases, steps, and methodological models. Mil. Med. Res..

[bib28] Xu P., Chen L., Zhang Z. (2021).

[bib29] Yang J., Li Y., Liu Q., Li L., Feng A., Wang T., Zheng S., Xu A., Lyu J. (2020). Brief introduction of medical database and data mining technology in big data era. J. Evid. Based. Med..

[bib30] Zhang Z., Chen L., Xu P., Hong Y. (2022). Predictive analytics with ensemble modeling in laparoscopic surgery: a technical note. Laparosc. Endoscopic Robotic Surg..

[bib31] Zhang Z., Liu J., Xi J., Gong Y., Zeng L., Ma P. (2021). Derivation and validation of an ensemble model for the prediction of agitation in mechanically ventilated patients maintained under light sedation. Crit. Care Med..

