# Supplementary material for: Developing an ensemble machine learning model for early prediction of sepsis-associated acute kidney injury
Source: iScience. 2022 Aug 12;25(9):104932. doi: 10.1016/j.isci.2022.104932 (PMC9429796; doi:10.1016/j.isci.2022.104932)
Supplement: Document S1. Figure S1–S9 and Tables S1–S7 [file mmc1.pdf]

iScience, Volume *25*

Supplemental information

Developing an ensemble machine learning model for early prediction of sepsis-associated acute kidney injury

Luming Zhang, Zichen Wang, Zhenyu Zhou, Shaojin Li, Tao Huang, Haiyan Yin, and Jun Lyu

The patient's stay_id, hadm_id, and subject_id were used as the patient's identification number to extract the patient's information.

When extracting the patient's apsiii score:

```
SELECT DISTINCT m1.*, mimic_derived.apsiii
INTO m2
FROM m1
LEFT OUTER JOIN mimic_derived.apsiii b
on m1.stay_id100= mimic_derived.stay_id
```

When extracting patient admission information

```
SELECT DISTINCT m2.*,mimic_core.admissions.*
into m3
from  m2
INNER JOIN mimic_core.admissions
ON m2.hadm_id100=mimic_core.admissions.hadm_id
```

When extracting the gender of the patients

```
SELECT DISTINCT m3.*,mimic_core.patients.gender
into m4
from m3 a
LEFT OUTER JOIN mimic_core.patients
ON m3.subject_id=mimic_core.patients.subject_id
```

Supplementary Figure S1 (Related to Data and code availability in STAR METHODS) SQL example for data extraction

Features	The 1st hour	The 2nd hour	The 3rd hour	...	The 11th hour	The 12th hour	Features summarize point
**Anion Gap**	12	15				13	13
**Creatinine**		2.0	1.8		2.5		2.5
**…**							…
**Heart rate**							NA
**SpO2**	96						96

Supplementary Table S1 (Related to Feature selection in STAR METHODS) An Example of Data Extraction for Individual Participants

Dataset	MIMIV-IV（Training)			eICU-CRD(Testing)			ZG(Testing)		
Hours to AKI	Total	AKI	Control	Total	AKI	Control	Total	AKI	Control
48	15,120	11,169	3,951	4,848	983	3,865	268	50	218
36	15,171	11,219	3,952	4,850	983	3,867	285	54	231
24	14,700	10,798	3,902	4,840	983	3,857	265	50	215
12	13,913	10,063	3,850	4,840	984	3,856	264	50	214

Supplementary Table S2 (Related to Feature selection in STAR METHODS) Size of Training and Testing Datasets for Prediction Models

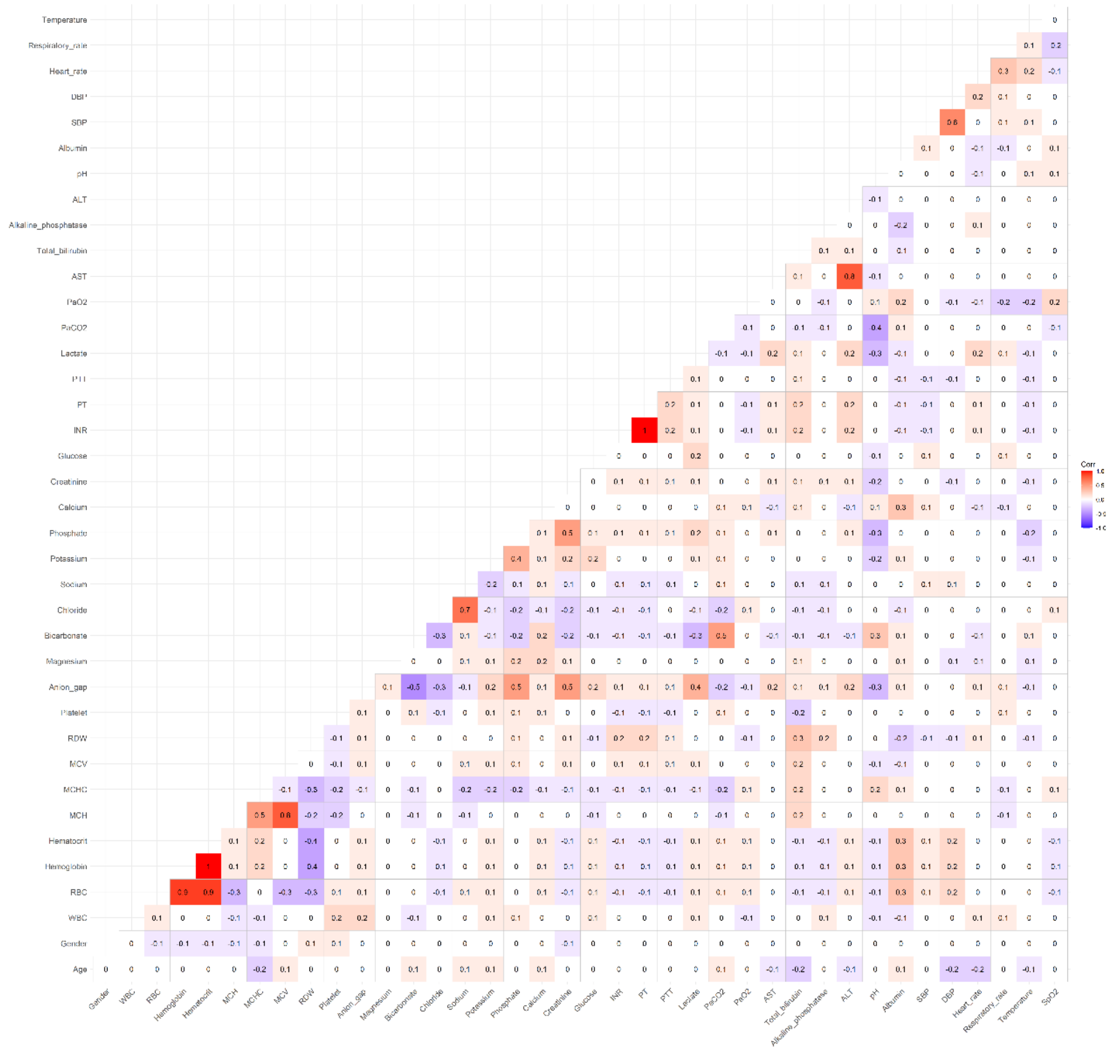

Supplementary Figure S2 (Related to Feature selection in STAR METHODS)
Heatmap of Person Correlations Between 38 Candidate Features

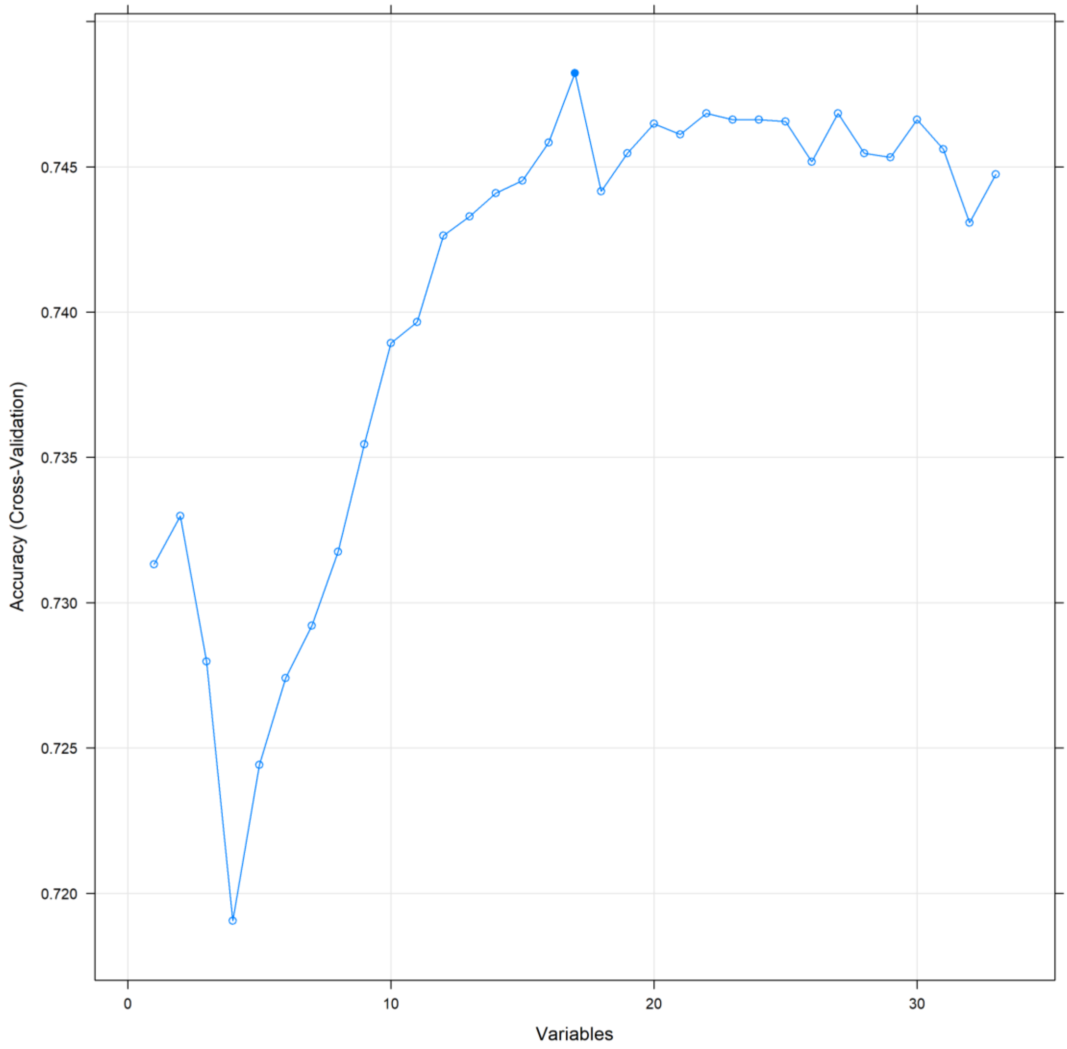

Supplementary Figure S3 (Related to Feature selection in STAR METHODS) The Result of Recursive Feature Elimination Based on Cross-validation of Random Forest

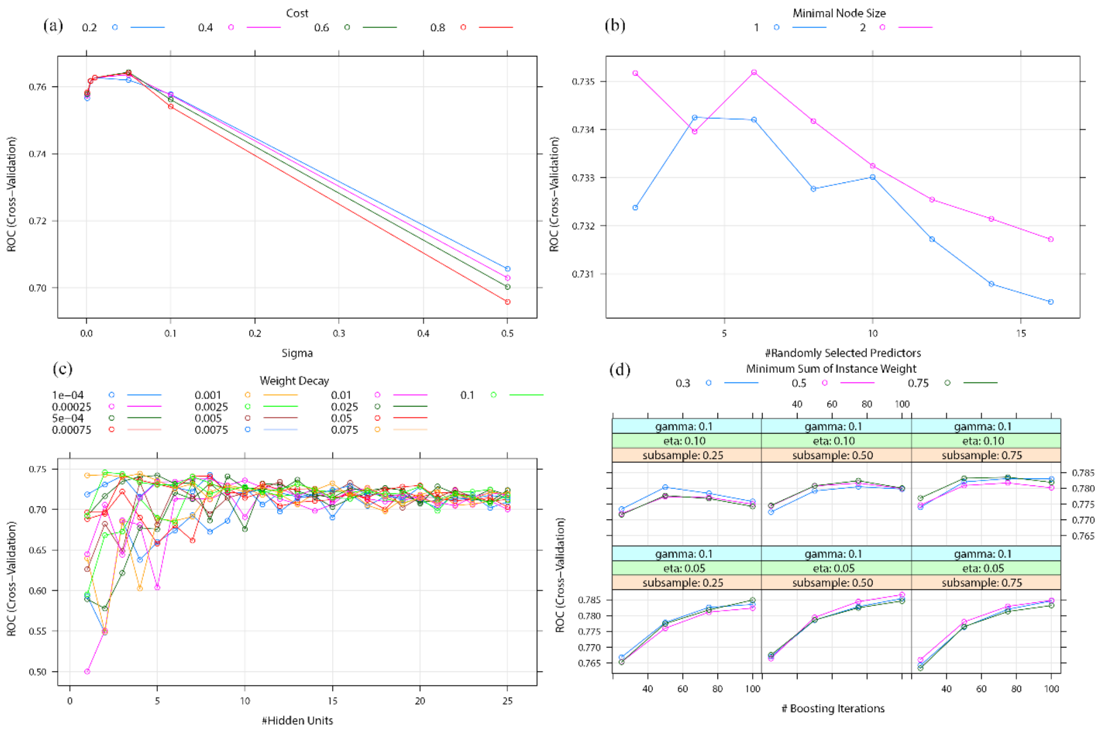

Supplementary Figure S4 (Related to Model Construction and Evaluation in STAR METHODS) Parameter Tuning of First-Level Learners for Ensemble Model Predicting AKI 12 Hours Before Onset Based On 5-fold Cross-Validation

(a) process of parameter tuning of SVM based on data 12 hours before AKI onset

(b) process of parameter tuning of RF based on data 12 hours before AKI onset

(c) process of parameter tuning of NNET based on data 12 hours before AKI onset

(d) process of parameter tuning of SVM based on data 12 hours before AKI onset

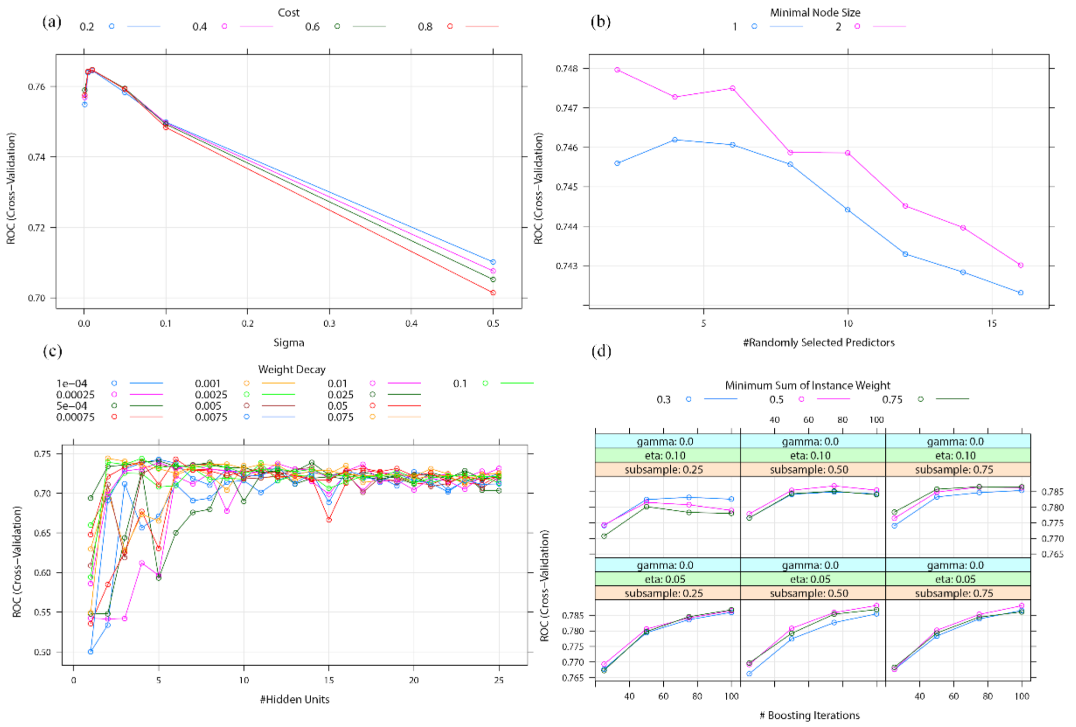

Supplementary Figure S5 (Related to Model Construction and Evaluation in STAR METHODS) Parameter Tuning of First-Level Learners for Ensemble Model Predicting AKI 24 Hours Before Onset Based On 5-fold Cross-Validation

(a) process of parameter tuning of SVM based on data 24 hours before AKI onset

(b) process of parameter tuning of RF based on data 24 hours before AKI onset

(c) process of parameter tuning of NNET based on data 24 hours before AKI onset

(d) process of parameter tuning of SVM based on data 24 hours before AKI onset

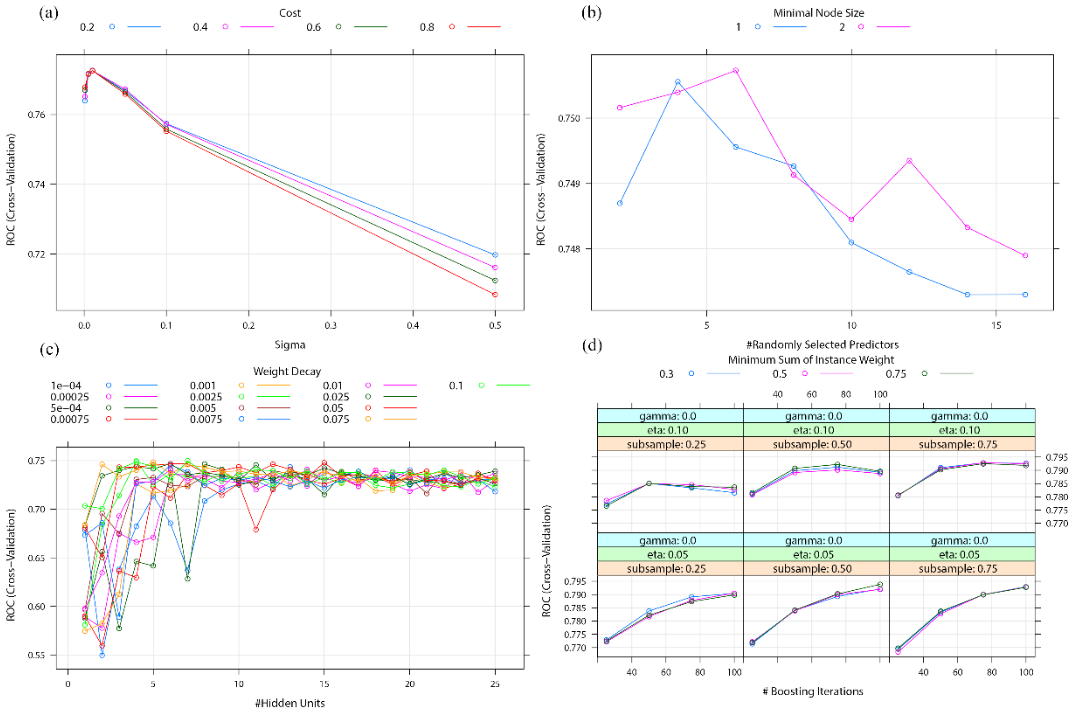

Supplementary Figure S6 (Related to Model Construction and Evaluation in STAR METHODS) Parameter Tuning of First-Level Learners for Ensemble Model Predicting AKI 36 Hours Before Onset Based On 5-fold Cross-Validation

(a) process of parameter tuning of SVM based on data 36 hours before AKI onset

(b) process of parameter tuning of RF based on data 36 hours before AKI onset

(c) process of parameter tuning of NNET based on data 36 hours before AKI onset

(d) process of parameter tuning of SVM based on data 36 hours before AKI onset

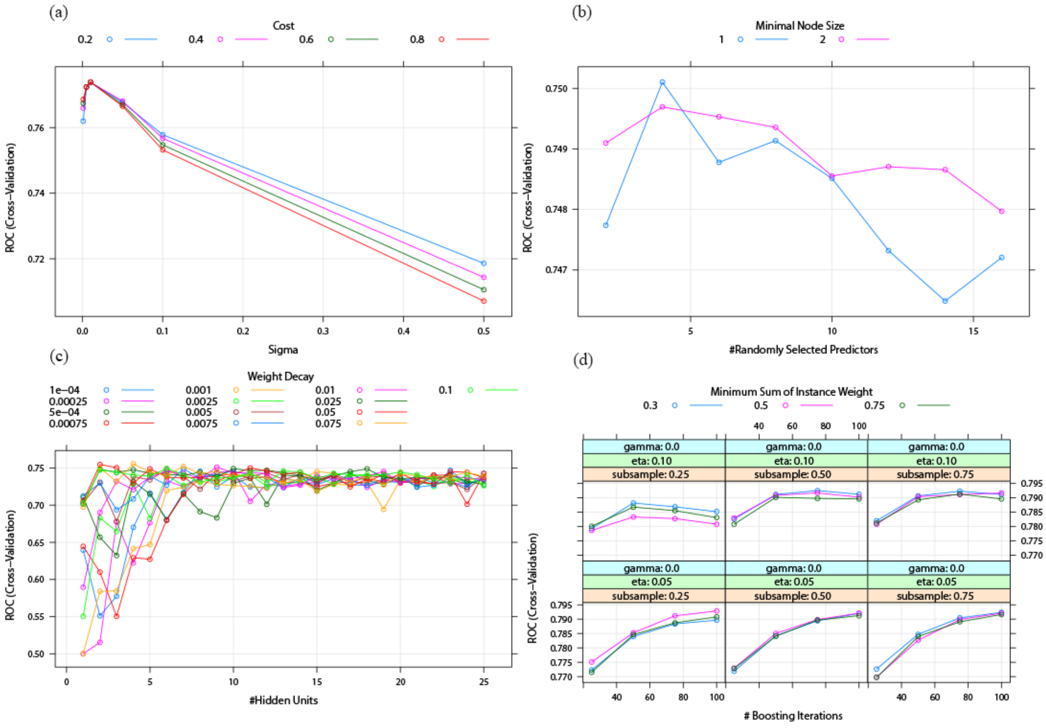

Supplementary Figure S7 (Related to Model Construction and Evaluation in STAR METHODS) Parameter Tuning of First-Level Learners for Ensemble Model Predicting AKI 48 Hours Before Onset Based On 5-fold Cross-Validation

(a) process of parameter tuning of SVM based on data 48 hours before AKI onset

(b) process of parameter tuning of RF based on data 48 hours before AKI onset

(c) process of parameter tuning of NNET based on data 48 hours before AKI onset

(d) process of parameter tuning of SVM based on data 48 hours before AKI onset

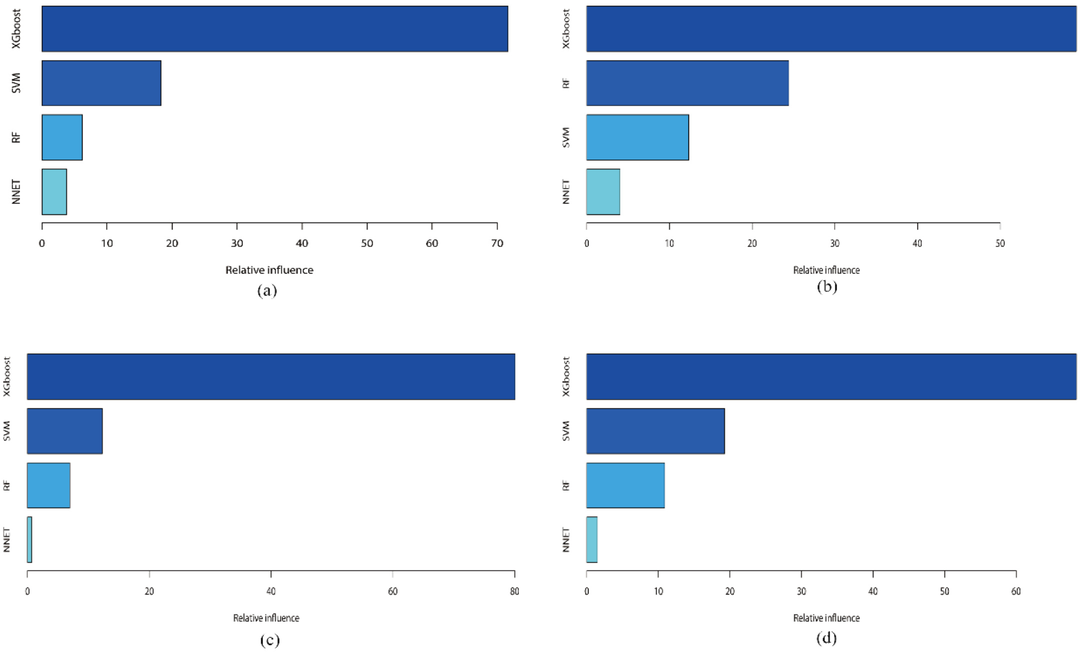

Supplementary Figure S8 (Related to Model Deployment in STAR METHODS**)**
Weighting of First-Level Learners for Ensemble Models

(a) The weighting of each first-level learner, including SVM, RF, NNET and XGboost for ensemble models 12 hours before AKI onset

(b) The weighting of each first-level learner, including SVM, RF, NNET and XGboost for ensemble models 24 hours before AKI onset

(c) The weighting of each first-level learner, including SVM, RF, NNET and XGboost for ensemble models 36 hours before AKI onset

(d) The weighting of each first-level learner, including SVM, RF, NNET and XGboost for ensemble models 48 hours before AKI onset

Algorithms	Hours to AKI			
	48	36	24	12
SVM	sigma=0.01, C=0.8	sigma=0.01, C=0.2	sigma=0.01, C=0.4	sigma=0.05, C=0.6
RF	mtry=4, splitrule='gini', min.node.size=1	mtry=6, splitrule='gini', min.node.size=2	mtry=2, splitrule='gini', min.node.size=2	mtry=6, splitrule='gini', min.node.size=2
NNET	size=4, decay=0.075	size=7, decay=0.1	size=2, decay=0.075	size=2, decay=0.1
XGboost	nrounds=100 max_depth=6, eta=0.05, gamma=0.1, colsample_bytree=1, min_child_weight=0.75, subsample=0.5	nrounds=100, max_depth=6 eta=0.05, gamma=0.1, colsample_bytree=1, min_child_weight=0.3, subsample-.5	nrounds=100, max_depth=6, eta=0.05, gamma=0.1, colsample_bytree=1, min_child_weight=0.5, subsample=0.75	nrounds=100, max_depth=6, eta=0.05, gamma=0.1, colsample_bytree=1, min_child_weight=0.5, subsample=0.5
Ensemble	n.trees=50, interaction.depth=3, shrinkage=0.1, n.minobsinnode=10	n.trees=50, interaction.depth=2, shrinkage =0.1, n.minobsinnode=10	n.trees=50, interaction.depth=7, shrinkage=0.1, n.minobsinnode=10	n.trees=50, interaction.depth=4, shrinkage=0.1, n.minobsinnode=10

Supplementary Table S3 (Related to Model Construction and Evaluation in STAR METHODS) The optimal hyperparameters for final models

Model Performance

Training

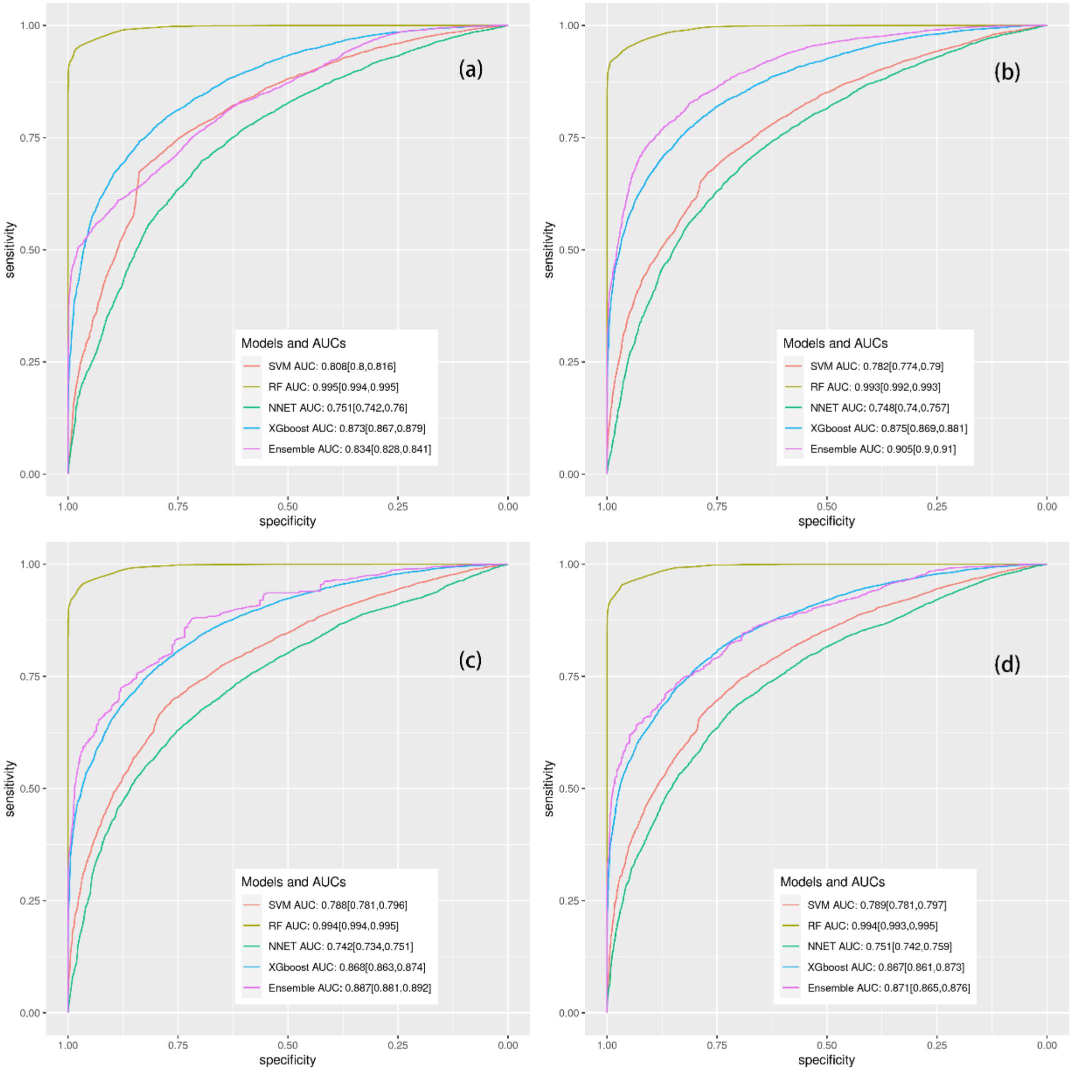

Supplementary Figure S9 (Related to Figure 3) Performance of Ensemble and Its First-Level Learners on the Training dataset

(a) Performance of model trained 12 hours before AKI onset

(b) Performance of model trained 24 hours before AKI onset

(c) Performance of model trained 36 hours before AKI onset

(d) Performance of model trained 48 hours before AKI onset

Hours to AKI	Sensitivity	Specificity	PPV	NPV	F1	Accuracy	Balanced Accuracy
eICU-CRD Database							
48	0.611	0.771	0.404	0.886	0.487	0.738	0.691
36	0.624	0.763	0.401	0.889	0.488	0.735	0.693
24	0.640	0.766	0.410	0.893	0.500	0.740	0.703
12	0.765	0.632	0.347	0.913	0.477	0.659	0.699
ZG Database							
48	0.660	0.771	0.398	0.908	0.496	0.750	0.715
36	0.630	0.762	0.382	0.898	0.476	0.737	0.696
24	0.520	0.874	0.491	0.887	0.505	0.808	0.697
12	0.780	0.617	0.322	0.923	0.456	0.648	0.698

Supplementary Table S4 (Related to Table 2) Evaluation Metrics of First level Learner-Support Vector Machines of the Ensemble Model

Hours to AKI	Sensitivity	Specificity	PPV	NPV	F1	Accuracy	Balanced Accuracy
eICU-CRD Database							
48	0.702	0.710	0.381	0.904	0.494	0.708	0.706
36	0.633	0.779	0.421	0.893	0.506	0.749	0.706
24	0.659	0.775	0.427	0.899	0.518	0.751	0.717
12	0.659	0.752	0.404	0.896	0.500	0.733	0.705
ZG Database							
48	0.820	0.596	0.318	0.935	0.458	0.638	0.708
36	0.722	0.719	0.375	0.917	0.494	0.719	0.720
24	0.780	0.740	0.411	0.935	0.538	0.747	0.760
12	0.760	0.743	0.409	0.930	0.532	0.746	0.752

Supplementary Table S5 (Related to Table 2) Evaluation Metrics of The First level Learner-RF of Ensemble Model

Hours to AKI	Sensitivity	Specificity	PPV	NPV	F1	Accuracy	Balanced Accuracy
eICU-CRD Database							
48	0.499	0.806	0.395	0.863	0.441	0.743	0.652
36	0.687	0.593	0.300	0.882	0.418	0.612	0.640
24	0.507	0.838	0.444	0.870	0.444	0.771	0.672
12	0.598	0.745	0.374	0.879	0.460	0.715	0.671
ZG Database							
48	0.800	0.564	0.296	0.925	0.432	0.608	0.682
36	0.741	0.688	0.357	0.919	0.482	0.698	0.715
24	0.720	0.754	0.405	0.921	0.518	0.747	0.737
12	0.880	0.617	0.349	0.957	0.500	0.667	0.748

Supplementary Table S6 (Related to Table 2) Evaluation Metrics of The First level Learner-NNET of Ensemble Model

Hours to AKI	Sensitivity	Specificity	PPV	NPV	F1	Accuracy	Balanced Accuracy
eICU-CRD Database							
48	0.698	0.718	0.387	0.903	0.498	0.714	0.708
36	0.709	0.723	0.394	0.907	0.507	0.720	0.716
24	0.693	0.769	0.434	0.908	0.533	0.754	0.731
12	0.721	0.710	0.388	0.909	0.504	0.712	0.715
ZG Database							
48	0.760	0.674	0.349	0.925	0.478	0.690	0.717
36	0.796	0.671	0.361	0.934	0.497	0.695	0.734
24	0.720	0.772	0.424	0.922	0.533	0.762	0.746
12	0.880	0.579	0.328	0.954	0.478	0.636	0.730

Supplementary Table S7 (Related to Table 2) Evaluation Metrics of The First level Learner-XGboost of Ensemble Model

Ensemble model Related to Model Deployment

..\model.rda
